# The effect of fecal microbial transplantation in a pediatric patient after 28 episodes of febrile urinary tract infection

**DOI:** 10.1007/s00467-025-06822-1

**Published:** 2025-05-20

**Authors:** Barbora Piteková, Ivan Hric, Eva Baranovičová, Jakub Zieg, Paul J. Planet, Viktor Bielik

**Affiliations:** 1 Department of Pediatric Emergency Medicine, National Institute of Children’s Diseases, Bratislava, Slovakia; 2 Department of Pediatric Urology, Faculty of Medicine, Comenius University and National Institute of Children’s Diseases, Bratislava, Slovakia; 3 Department of Pediatrics, Slovak Medical University in Bratislava, Bratislava, Slovakia; 4 Biomedical Center, Institute of Clinical and Translational Research, Slovak Academy of Sciences, Bratislava, Slovakia; 5 Biomedical Center Martin, Jessenius Faculty of Medicine in Martin, Comenius University in Bratislava, 036 01 Martin, Slovakia; 6 Department of Pediatrics, Second Faculty of Medicine, Charles University and Motol University Hospital, Prague, Czech Republic; 7 Children´S Hospital of Philadelphia, Philadelphia, PA USA; 8 Department of Pediatrics, University of Pennsylvania, Philadelphia, PA USA; 9 Department of Biological and Medical Sciences, Faculty of Physical Education and Sport, Comenius University, Bratislava, Slovakia

**Keywords:** Fecal microbial transplantation, Gut microbiome, Metabolome, Recurrent pyelonephritis, Chronic kidney disease

## Abstract

Recurrent febrile urinary tract infections (fUTIs) in children can lead to serious complications such as renal scarring and progressive chronic kidney disease (CKD), with growing evidence indicating that gut microbiome dysbiosis may play a key role in their development. Fecal microbial transplantation (FMT) is an established therapeutic approach for restoring gut microbial balance; however, its use in patients with recurrent fUTIs remains limited and underexplored. This case study describes a 10-year-old boy with recurrent fUTIs and CKD secondary to a posterior urethral valve (PUV) anomaly. The patient was administered a total of seven doses of FMT. FMT reduced pathogenic *Enterobacteriaceae*, increased beneficial short-chain fatty acid (SCFA)-producing genera, and correspondingly raised SCFA levels, indicating restoration of gut microbiota balance. FMT presents an innovative therapeutic option for pediatric patients with recurrent fUTIs, demonstrating outstanding clinical outcomes.

## Introduction

Recurrent fUTIs represent a significant clinical challenge, frequently resulting in multiple hospitalizions and extensive medical interventions. Furthermore, recurrent fUTIs may lead to significant consequences such as renal scarring, arterial hypertension, and progressive CKD [1]. All of these features were present in our patient. The current therapeutic strategy in the prevention of fUTI recurrence is the administration of long-term antibiotic prophylaxis [1]. However, this strategy is not efficient, and over time, it leads to the emergence of multidrug-resistant uropathogens [1]. There has also been a strong, documented connection between the gut microbiome and fUTI. Children with fUTI have a higher abundance of *Enterobacteria* in stool samples [2].

This article aims to enhance the existing evidence for microbiome-based therapeutics in the management of recurrent fUTIs by outlining the clinical course, microbiome analysis, and outcomes related to FMT in the child with recurrent fUTIs due to PUV.

## Case study

A 10-year-old boy with a history of recurrent fUTIs, totaling 28 episodes, and CKD, stage 3 due to PUV was referred to our tertiary center. The patient was prenatally suspected of having cystic involvement of both kidneys. Postnatally, he was diagnosed with bilateral hydroureteronephrosis. Due to the postnatal progression of renal parameters, bilateral puncture nephrostomies were introduced in the second week of life, and the patient developed the first urosepsis. In the fourth week of life, posterior urethral valve dissection was performed, and bilateral ureterostomies were placed for urinary diversion. Various urinary tract interventions were implemented, including vesicostomy at the age of 4 to 7 years (2018–2021), at the age of 7 to 8 years coiling (2021–2022), and from age 8 years to the present day epicystostomy (from 2022 onward). However, the interventions and continuous prophylactic antibiotics did not prevent recurrent fUTIs. In total, our patient experienced 28 fUTI episodes. Each episode of fUTI presented as urosepsis and required intravenous administration of broad-spectrum antibiotics due to the multidrug-resistant nature of the uropathogens.

Recurrent fUTIs resulted in progressive kidney scarring, reduction of glomerular filtration rate, and development of stage 3 chronic kidney disease (the last dimercaptosuccinic acid (DMSA) scans showed 5% of residual function in the right kidney). Consequently, the patient was referred for nephrectomy of the affected kidney. However, the procedure was deferred, and the patient was instead referred for experimental FMT.

## Methods

### FMT procedure

The FMT donor was a known healthy adult subject, regularly tested with a well-defined microbiome profile. FMT was delivered using the high enema method with a high rectal tube. The first round of FMT was administered in August 2023, with one dose given every 3 days. As the patient developed another episode of fUTI caused by *E. coli*, we proceeded with retransplantation in September 2023. In April 2024, one additional dose of maintenance FMT was given. In total, the patient received seven doses of FMT. Simultaneously, the patient was put on a diet supporting a healthy microbiome (at least three meals per day) and received daily prebiotics.

### The microbiome and metabolome analysis

The patient had a stool and urine sample collected before FMT administration and monthly after FMT administration, from August 2023 to August 2024 (in total, 12 months). Microbiota composition was analyzed monthly using 16S rRNA sequencing, and the concentrations of 21 metabolites in stool samples were analyzed using nuclear magnetic resonance spectroscopy. Details of this data processing can be found in Adamová et al. [3]. We used the Mann–Kendall test to evaluate trends in microbial genera and SCFA levels post-FMT.

## Clinical course after FMT

**Fig. 1:**
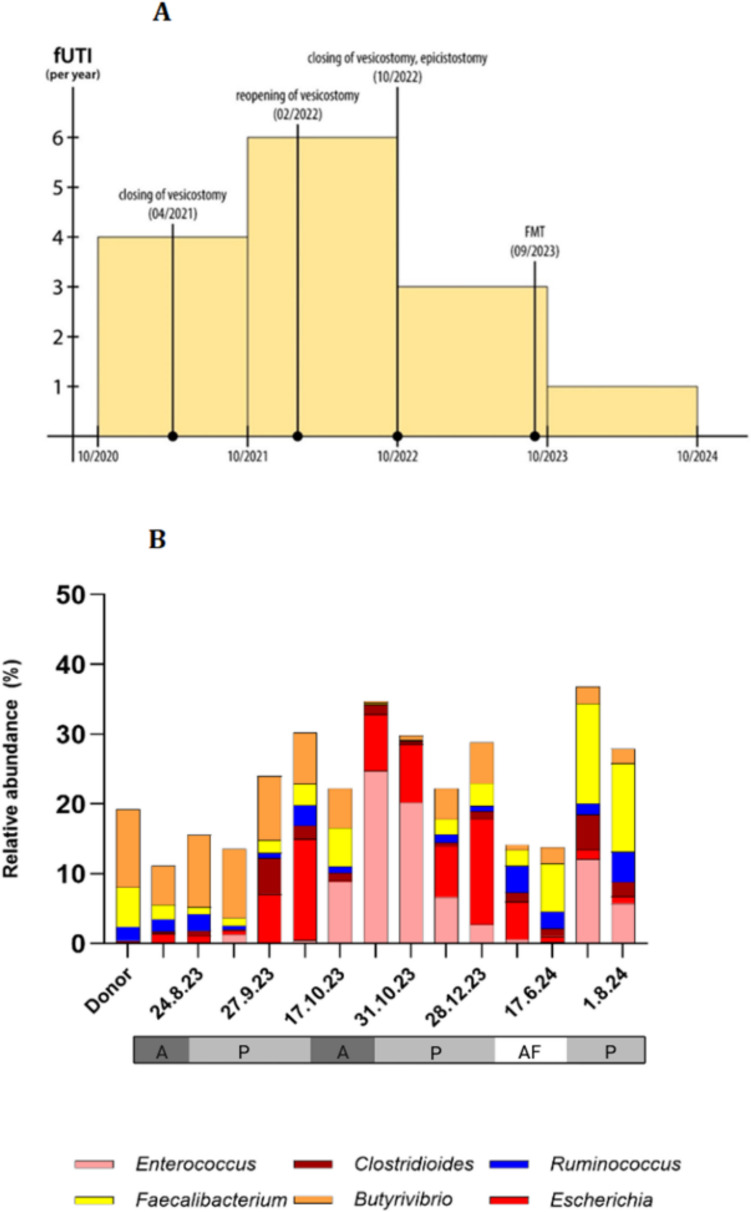
Clinical and microbiological outcomes following FMT. **A** Number of fUTIs per year from October 2020 to October 2024, demonstrating a complete absence of fUTIs after FMT administration. **B** Relative abundance of pathogenic (*Escherichia coli*) and probiotic (*Faecalibacterium*, *Ruminococcus*) genera in the patient’s gut microbiota over time following FMT, showing a decline in pathogenic bacteria and an increase in beneficial SCFA-producing genera. A, antibiotics; P, prophylactic antibiotics; AF, antibiotic-free period

The patient had his last episode of fUTI at the age of 9 years and 2 months (in October 2023). The reduction in fUTI episodes after FMT administration is clearly shown in Fig. 1A. Currently, the patient has been without a new fUTI for 12 months (from October 2023 to October 2024).

## Microbiome and metabolome analyses

Before FMT, our analysis of the gut microbiota revealed a high abundance of pathogenic *Enterobacteriaceae*, particularly *Escherichia coli* (*E. coli*) (14.42%), and a low abundance of the probiotic *Faecalibacterium* (3.12%). From November 2023, a notable reduction in *E. coli* abundance was observed after FMT, decreasing to less than 2%, alongside a significantly increasing trend in *Faecalibacterium* to 9.84% (*p* = 0.02) (Fig. 1B). Similarly, metabolomic analysis showed suppressed relative concentrations of butyrate before FMT.

From November 2023, SCFA-producing genera showed an increasing trend (*p* = 0.02; Fig. 1B). The relative concentrations of SCFAs also improved, with a significant upward trend in propionate (*p* = 0.03).

## Discussion

The main finding of this study is the successful modulation of gut microbiota composition following FMT, characterized by a reduction in pathogenic *Enterobacteriaceae* (particularly *E. coli*) and a concurrent increase in beneficial SCFA-producing bacteria, including *Faecalibacterium, Blautia,* and *Ruminococcus*. Furthermore, we observed a substantial enhancement in microbial metabolic activity, demonstrated by elevated relative concentrations of butyrate. These results underscore the therapeutic potential of FMT in restoring microbiota balance and improving microbial metabolism, thus contributing to improved clinical outcomes.

The gut serves as the primary reservoir for UTI pathogens, and disturbances in the gut microbiome have been suggested to contribute to the development of fUTIs [2, 4, 5]. In our patient, we observed a high abundance of *Enterobacteriaceae* in stool samples, consistent with previous findings [2], along with a notably low level of butyrate-producing bacteria. Administration of FMT led to a significant reduction in *E. coli* abundance and a marked increase in beneficial probiotic genera. We propose that this microbiome modification was the principal factor underlying the subsequent absence of fUTI episodes in our patient.

The role of FMT in the management of recurrent fUTIs remains unclear, particularly in the pediatric population, with available data limited to a single case report [4]. In adults, however, FMT has demonstrated efficacy in reducing fUTI episodes and improving microbial antibiotic susceptibility [5]. Kidney scarring, often a consequence of recurrent fUTIs, is notoriously difficult to reverse with medical interventions, as evidenced by the progression of kidney damage in our patient’s right kidney on DMSA scans. Based on our experience, we propose that early administration of FMT, following only a few episodes of fUTI, could serve as a preventive strategy against renal scarring.

In conclusion, FMT may represent an effective new therapeutic option for patients with recurrent fUTIs. Although current pediatric data are limited, our case supports the critical role of gut microbiome composition in fUTI recurrence. This study not only highlights the potential of microbiome-targeted therapies in pediatric nephrology but also provides a strong rationale for larger clinical trials to validate this promising approach.

## Summary

### What is new?

This study provides the first pediatric evidence that fecal microbial transplantation (FMT) can effectively prevent recurrent febrile urinary tract infections (fUTIs) by restoring gut microbiota balance and improving metabolome profiles.

## Data Availability

All data generated or analyzed during this study are included in this published article.

## Abbreviations

CKD: Chronic kidney disease
DMSA: Dimercaptosuccinic acid
*E. coli*: *Escherichia coli*
FMT: Fecal microbial transplantation
fUTI: Febrile urinary tract infection
PUV: Posterior urethral valve
SCFA: Short-chain fatty acid

## References

[1] Williams G, Craig JC (2019) Long-term antibiotics for preventing recurrent urinary tract infection in children. Cochrane Database Syst Rev 4:CD001534. 10.1002/14651858.CD001534.pub4 30932167 10.1002/14651858.CD001534.pub4 PMC6442022

[2] Paalanne N, Husso A, Salo J et al (2018) Intestinal microbiome as a risk factor for urinary tract infections in children. Eur J Clin Microbiol Infect Dis 37:1881–1891. 10.1007/s10096-018-3322-7 30006660 10.1007/s10096-018-3322-7

[3] Adamová LM, Slezáková D, Hric I et al (2024) Impact of dance classes on motor and cognitive functions and gut microbiota composition in multiple sclerosis patients: randomized controlled trial. Eur J Sport Sci 24:1186–1196. 10.1002/ejsc.12166 38967986 10.1002/ejsc.12166 PMC11295098

[4] Vedrik KEW, de Meij TGJ, Bokenkamp A et al (2022) Transmission of antibiotic-suspectible Escherichia coli causing urinary tract infections in a fecal microbiota transplantation recipient: consequences for donor screening? Open Forum Infect Dis 9:ofac324. 10.1093/ofid/ofac324 35899275 10.1093/ofid/ofac324 PMC9314704

[5] Lagier JC, Raoult D (2020) Faecal microbiota transplantations and urinary tract infections. Lancet 395:270–271. 10.1016/S0140-6736(19)32992-7 31982066 10.1016/S0140-6736(19)32992-7

